# The Effects of Cooperative and Competitive Situations on Statistical Learning

**DOI:** 10.3390/brainsci12081059

**Published:** 2022-08-10

**Authors:** Yajie Si, Xinyu Chen, Wei Guo, Biye Wang

**Affiliations:** 1 College of Physical Education, Yangzhou University, Yangzhou 225009, China; 2 Institute of Sports, Exercise and Brain, Yangzhou University, Yangzhou 225127, China

**Keywords:** cooperation situation, competition situation, general learning, statistical learning

## Abstract

Devising cooperative or competitive situations is an important teaching strategy in educational practices. Nevertheless, there is still controversy regarding which situation is better for learning. This study was conducted to explore the effects of cooperative and competitive situations on statistical learning, through the alternating serial reaction time (ASRT) task. Individual cooperative and competitive situations were devised in this study, in which individual situation served as the control condition. Ninety recruited participants were randomly assigned to a cooperative, competitive, or individual group to perform the ASRT task. For general learning, cooperative and competitive situations could indeed make learners respond faster, and there was no significant difference in the RT between the cooperative and competitive groups. Moreover, statistical learning was observed in all three groups. An additional analysis of the early stage of the experiment showed that the learning effect of the competitive group was greater than those of the cooperative and individual groups, in terms of statistical learning. However, the final learning effect was not significantly different among the three groups. Overall, the cooperative and competitive situations had a positive impact on learning and enabled the students to acquire approximately the same learning effect in a shorter time period, compared with the individual situation. Specifically, the competitive situation accelerated the statistical learning process but not the general learning process.

## 1. Introduction

It is a common teaching strategy to create a cooperative or competitive situation in educational practice [1,2,3,4]. In cooperative situations, students work together in groups to achieve a common goal [5,6], for example, the pursuit of higher total scores [7]. In competitive situations, students need to compete against others to achieve their learning objectives in order to acquire higher achievement [8]. It is considered that cooperation and competition can encourage students to be more focused and think more positively so that they can actively participate in their own knowledge construction and improve their academic performance [4,8,9].

Studies have found that the learning performance in a cooperative or competitive situation is superior to that in an individual situation [10,11,12]. However, when the two learning situations were compared simultaneously, contradictory results were obtained. Some studies have demonstrated that both cooperation and competition promote learning performance, with no significant difference [8,13], while others have concluded that cooperation is superior to competitive and individual learning situations [14,15]. Notwithstanding, previous studies have mainly focused on the learning of declarative knowledge (e.g., vocabulary memory, mathematics, etc.), but few studies have concentrated on statistical learning in situations of cooperation and competition, which are subcomponents of procedural learning [16,17,18].

Statistical learning [19] refers to the process or learning patterns of extracting probabilistic (frequency) structures from the environment [16,20,21,22,23]. This process unfolds over time and usually occurs unconsciously [24]. Statistical learning is a robust mechanism of the brain, which is crucial in the perceptual and cognitive domains [25]. Statistical learning plays an indispensable role in perception [25,26], associative learning, predictive processing [25,27], and the learning of rule-based skills [24,28], such as spoken language, music, or motor skills [24,29,30]. For example, when playing a piano piece, notes that co-occur more frequently in the music score can be predicted by statistical learning. Therefore, combinations that are executed more frequently are likely to be executed faster. Statistical learning is a fundamental learning mechanism and seems to be present at birth [19,31,32,33]. People use statistical learning to master the laws of probability in the outside world so that they can more effectively predict upcoming events and prepare for action [32,34,35,36].

To explore statistical learning, an alternating serial reaction time (ASRT) task, developed by Howard, has been used by many researchers [16,17,37]. The ASRT task paradigms—with random, probabilistic, and deterministic transformations—are thought to be more dependent on implicit learning mechanisms because the repeated sequences are more complex and hidden than they are in the classic finger-tapping task [38]. The ASRT task has been shown to be effective for studying implicit sequence learning, such as general and statistical learning [16,17,36,37,39,40]. General skill learning is reflected in the overall response time (RT) in the ASRT tasks [16,21,40,41], while statistical learning is a basic implicit learning mechanism, which requires learners to extract probability regularities from the sequences [16,21,23]. ASRT tasks can also be combined with neuroelectrophysiological techniques to explore the neural mechanisms of statistical learning [40,42,43].

Previous studies have mainly focused on the impact of cooperation and competition on declarative knowledge learning, but there are few studies that have explored their effects on statistical learning. Therefore, this pilot study was conducted by setting up three different situations, aiming to explore the effects of cooperative, competitive, and individual situations on statistical learning through the ASRT task.

## 2. Materials and Methods

### 2.1. Participants

Ninety undergraduate or graduate students were recruited through an announcement posted on the campus of Yangzhou University, China. All participants were randomly divided into a cooperative, competitive, or individual group. The participants in all groups were paired so that every two participants were of the same gender, in order to overcome the potential impact of gender differences [44,45]. Three participants failed to complete the experiment, so the paired participants were forced to terminate the experiment, causing six participants to be excluded. Another six participants were excluded due to a low testing accuracy. A total of 12 participants were excluded from the experiment, and 78 were included in the analysis. There were 27 participants (18 males and 9 females, *M_age_* = 21.19 years old, *SD_age_* = 0.9 years) in the cooperative group; 24 participants (10 males and 14 females, *M_age_* = 21.54, *SD_age_* = 1.7) in the competitive group; and 27 participants (13 males and 14 females, *M_age_* = 22.15, *SD_age_* = 1.7) in the individual group. All participants were right-handed and had normal or corrected-to-normal vision. They had no developmental, neurological, or psychiatric disorders and signed a written informed consent form prior to the experiment. The study was approved by the local ethics committee and was carried out in accordance with the approved guidelines. Each participant was paid RMB 30 for completing the experiment.

### 2.2. Design

This study had a 4 × 3 mixed-factorial design. The within-subject factor was epoch (epoch 1 vs. epoch 2 vs. epoch 3 vs. epoch 4), and the between-subject factor was group (individual group vs. cooperative group vs. competitive group).

### 2.3. ASRT Task

The ASRT task [37,46] was programmed in Matlab, with the psychtoolbox [47]. In a typical trial, one of the four empty squares, arranged horizontally on a gray screen, was filled with the color red (target stimulus). The stimulus remained visible until the participant pressed the corresponding key. The inter-stimulus interval was set to 120 ms [38,46]. The sequence order of the four possible positions (encoded as 1, 2, 3, and 4) of the target stimulus was determined by a sequence of eight elements (e.g., 3r1r2r4r, where the number represents the position of the four squares on the screen, and r represents events randomly selected from four possible positions) [21,37,38,42,46].

All participants needed to complete a total of 20 blocks, with 85 trials per block. The first five stimuli of each block were presented randomly (excluded for analysis) for practice, followed by an eight-element alternating sequence, repeated ten times [23,39,41,48]. According to the permutation rules, six unique sequences of predetermined elements (e.g.,1r2r3r4r, 1r2r4r3r, …, and 4r3r2r1r) were created. The participants were randomly assigned a pseudo-random sequence [21,23,41].

**Figure 1:**
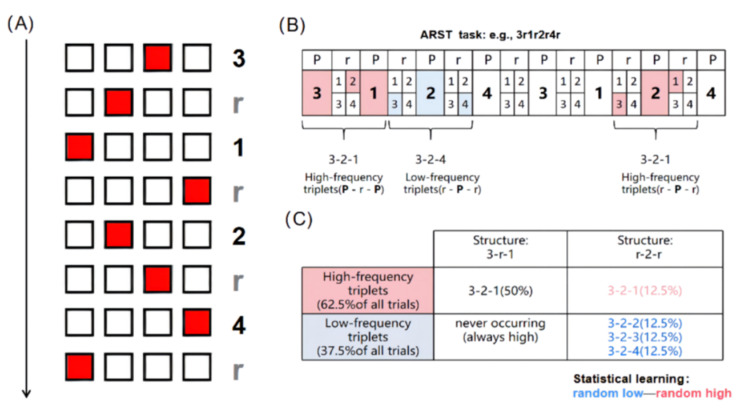
Paradigm of the alternating serial reaction time (ASRT) task (refer to [18,40]). (**A**) ASRT task: four squares were arranged horizontally in the center of the screen, with one of the squares filled with red as the target stimulus. The sequence order of the four possible positions (encoded as 1, 2, 3, and 4, respectively) of the target stimulus was determined by a sequence of eight elements (e.g., 3r1r2r4r, where the number represents the position of the four squares on the screen, and r represents events randomly selected from four possible positions). The stimulus remained visible until the participant pressed the corresponding key, and the inter-stimulus interval was 120 ms. (**B**) In the alternating sequence structure, some runs of three consecutive elements (called triplets) occurred more frequently (pink) than others (blue). In the sequence example shown (3-r-1-r-2-r-4-r), the triplet of r-1-r, r-2-r, r-4-r, or r-3-r is called a random triplet because it includes two random elements (the first and third elements) and only one pattern element (the second element) in the middle. (**C**) In the example shown, the triplet 3-r-1 has a probability of 62.5% and is called a high-frequency triplet (pink). The probability of occurrence of a pattern triplet is 50%; therefore, it is called a pattern high-frequency triplet. In a random triplet (r-2-r), the triplet 3-2-1 has a probability of 12.5%, so it is called a random high-frequency triplet (pink). In contrast, triplets such as 3-2-2, 3-2-3, and 3-2-4 have a low probability (12.5%) of occurring and are called random low-frequency triplets (blue), because they can only occur when the third element of the triplet is random. Statistical learning is defined as a faster response to random high-frequency triplets than to random low-frequency triplets.

As shown in Figure 1A, the pattern sequence elements alternated with random elements in the ASRT task. This alternating sequence structure generated some triplets consisting of three consecutive elements, which appeared more frequently than others [36,37,42,48]. Depending on the type (pattern and random) and frequency (high and low) of each element in each triplet, three different conditions—including pattern high-frequency, random high-frequency, and random low-frequency triplets—were constructed [36]. As shown in the example sequence (3r1r2r4r) of Figure 1B, the triplets 3-r-1, 1-r-2, 2-r-3, and 3-r-2 are called pattern triplets because they have two pattern elements (the first and third elements). These two elements appear regularly in the triplet, with only one random element (the second element) between the two pattern elements. In contrast, r-1-r, r-2-r, r-4-r, and r-3-r triplets are called random triplets because they include two random elements (the first and third elements), with only one pattern element (the second element) in the middle. As shown in Figure 1C, the occurrence probability of the triplet starting with element “3” and ending with element “1” is 62.5% and is called a high-frequency triplet. The probability of occurrence of the pattern triplet is 50%; therefore, it is called a pattern high-frequency triplet. In a random triplet, the triplet that starts with element “3” and ends with element “1” has a probability of 12.5%, so it is called a random high-frequency triplet. That is to say, high-frequency triplets can be composed of both pattern elements and random elements. In contrast, triplets such as 1-X-3 or 4-X-2 have a low probability (12.5%) of occurring because they can only occur when the third element of the triplet is random. Since their probability of occurring is lower (12.5% × 3 = 37.5%) [21,36,39,49], they are referred to as random low-frequency triplets. Of the 64 possible triplets, high-frequency triplets are approximately five times more likely to occur than low-frequency triplets [36]. Previous studies have shown that people implicitly learn triplet frequencies and respond more quickly and accurately to high-frequency triplets than to low-frequency triplets, despite the fact that they are unaware of the alternating nature of the sequence [50].

### 2.4. Procedure

Each pair of participants was arranged in a dimly lit room. The two participants were separated by an opaque, white partition and were required to be quiet during the experiment. Each participant sat comfortably, approximately 60 cm in front of a computer screen. The screen presented instructions telling the participant to press the space bar to start the experiment. The participants were required to press the corresponding keys on a computer keyboard (Z, X, N, and M on a QWERTY keyboard) as quickly and accurately as possible. The participants were instructed to respond to the following rules: Z, left middle finger; X, left index finger; N, right index finger; and M, right middle finger (as shown in Figure S1 in the Supplementary Materials). There were 20 blocks in total for the ASRT task. All participants were asked to rest for at least 20 s before starting the next block. The whole process took approximately 20 min.

**Figure 2:**
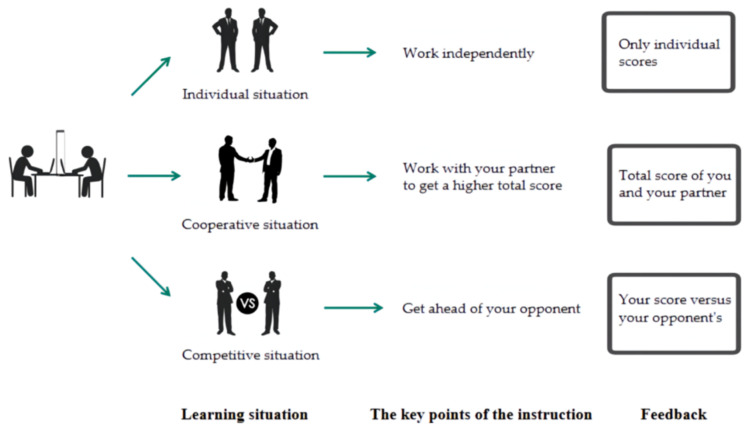
The description of the experimental procedure.

The participants in all three groups were required by the instructions to respond as quickly and accurately as possible (detailed experimental task and situation instructions were provided in the Supplementary Materials). In the cooperative situation, each pair of participants was a partnership and was asked to work together to improve the total score of both of them, which was composed of the RT and accuracy. Participants in the cooperative group only received on-screen feedback on the total scores of the pair between blocks, they did not know their partners’ scores. In the competitive situation, the pair were opponents The participants were asked to try hard to surpass their opponent’s score, they received feedback on scores of both their own and their opponent’s. Our program ensured that the paired participants in the cooperative and competitive groups started the next block at the same time. In the individual situation, the pair was not given instructions about cooperation or competition. They were asked to complete the task on their own and focus on improving their own scores, they only received feedback on their own scores (Figure 2). The feedback was provided in text form on the screen.

### 2.5. Statistical Analysis

A total of 20 blocks were average, collapsed into four epochs [18,21,38,41] to reveal the progress of the learning process. The median RT for each epoch of each participant was calculated separately [21,49,51]. If a triplet included two random elements (the first and third elements) and only one pattern element (the second element) in the middle, it was defined as a random triplet. It was defined as a high-frequency triplet if it was, more or less, predictable from the first element [42]. Triplets with a low frequency of forming repetitions (e.g., 333) or trills (e.g., 323) were excluded from the analysis because the participants generally showed a pre-existing tendency to respond to them [37,42,48,49,52,53], and the trials in which the participants pressed the wrong key were also discarded.

The sequence structure of the ASRT task allowed general learning and statistical learning to be calculated. General learning was defined as a decrease in the median RT for each subsequent epoch over the whole learning process [16,21,40,41]. A 4 × 3 mixed-design analysis of variance (ANOVA) was conducted on the median RT for each epoch of the individual group, cooperative group, and competitive group. The within-subject factor was epoch (4: epoch 1 vs. epoch 2 vs. epoch 3 vs. epoch 4), and the between-subject factor was group (3: individual group vs. cooperative group vs. competitive group).

The learning effect of general learning was calculated as the median RT of the first epoch, minus that of the fourth epoch. One-way ANOVA was conducted on the general learning effect of the three groups. The dependent variable was the general learning effect, and the independent variable was the group.

Statistical learning was defined as a faster response to random high-frequency triplets than to random low-frequency triplets [16,17,18,36,37,39,40,48]. A 4 × 2 × 3 mixed-design ANOVA was conducted on the median RT for each random high-frequency triplet and random low-frequency triplet, in each epoch of the three groups. Within-subject factors were epoch (4: epoch 1 vs. epoch 2 vs. epoch 3 vs. epoch 4) and frequency (2: high vs. low). The between-subject factor was group (3: individual group vs. cooperative group vs. competitive group).

The learning effect of statistical learning was calculated as the median RT of the random low-frequency triplets, minus that of the random high-frequency triplets [17,36,37]. A 4 × 3 mixed-design ANOVA was conducted on the median RT of each epoch in the three groups. The within-subject factor was epoch (4: epoch 1 vs. epoch 2 vs. epoch 3 vs. epoch 4), and the between-subject factor was group (3: individual group vs. cooperative group vs. competitive group).

## 3. Results

### 3.1. General Skill Learning

**Figure 3:**
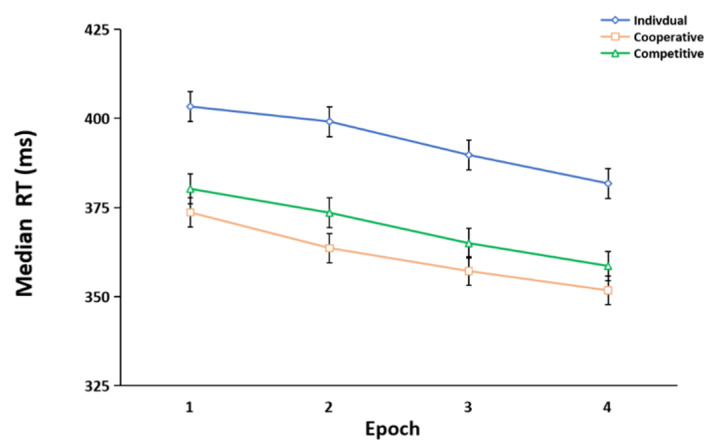
Median reaction times (RTs) of the individual group, cooperative group, and competitive group across the learning process (epochs 1–4). The error bars indicate the standard error.

The median RTs of the four epochs for each group, analyzed by ANOVA, are shown in Figure 3. The results of ANOVA revealed a significant main effect for epoch, *F* (3, 225) = 42.120, *p* < 0.001, $\eta_{p}^{2}$ = 0.360. The RT decreased with epoch. Post-hoc comparisons of the epochs revealed significant differences in the RT between epoch one and epoch four. The main effect of the group was also significant, *F* (2, 75) = 4.401, *p* = 0.016, $\eta_{p}^{2}$ = 0.105. Post-hoc comparisons showed that the RTs of the cooperative group (*p* = 0.006) and the competitive group (*p* = 0.04) were faster than those of the individual group, but there was no significant difference between the cooperative group and the competitive group (*p* = 0.501). In addition, the interaction between epoch and group was not significant, *F* (6, 225) = 0.298, *p* = 0.937, $\eta_{p}^{2}$ = 0.008.

**Figure 4:**
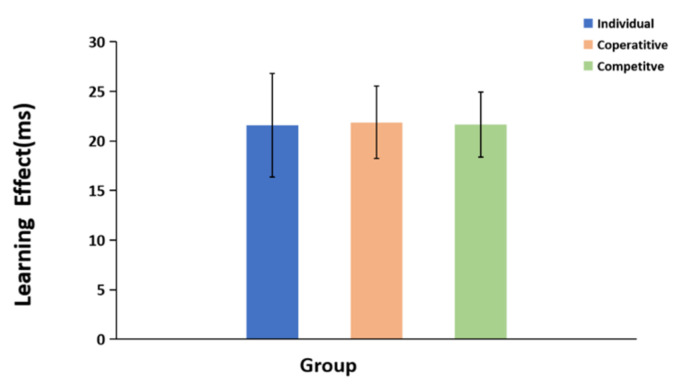
The general learning effects for the individual group, cooperative group, and competitive group. The error bars indicate the standard error.

The general learning effect was calculated as the median RT of the first epoch, minus that of the fourth epoch and is shown in Figure 4. The general learning effect for each group was analyzed by one-way ANOVA. The results showed that the main effect of the group was not significant, *F* (2, 75) = 0.154, *p* = 0.999, *η*^2^ < 0.001, demonstrating that the situation of cooperation or competition had no effect on the general learning effect for each group.

### 3.2. Statistical Learning

**Figure 5:**
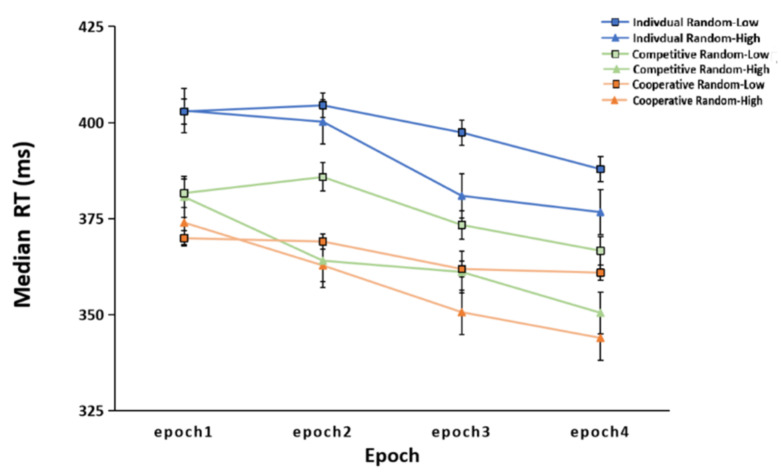
Median reaction times (RTs) of random low-frequency triplets and random high-frequency triplets in the individual group, cooperative group, and competitive group, across the learning process (epochs 1–4). The error bars indicate the standard error.

The median RTs of the random low-frequency triplets and the random high-frequency triplets for the four epochs in each group, analyzed by ANOVA, are shown in Figure 5. The results of the ANOVA revealed a significant main effect for frequency (*F* (1, 75) = 40.378, *p* < 0.001, $\eta_{p}^{2}$ = 0.350), indicating a faster response time to random high-frequency triplets than to random low-frequency triplets. The main effect of epoch was significant (*F* (3, 225) = 25.815, *p* < 0.001, $\eta_{p}^{2}$ = 0.256). Post-hoc comparisons of the epochs suggested that there was no significant difference in the RTs between epoch one and epoch two (*p* = 0.114) and that the RTs of epoch three and epoch four were significantly faster than those of epoch one and epoch two; however, the RT of epoch four was significantly faster than that of epoch three (*p* = 0.003). The main effect of the group was also significant (*F* (2, 75) = 4.622, *p* = 0.013, $\eta_{p}^{2}$ = 0.110). Post-hoc comparisons showed that the RTs of the cooperative group (*p* = 0.004) and the competitive group (*p* = 0.041) were faster than that of the individual group, but there was no significant difference between the RTs of the cooperative group and the competitive group (*p* = 0.441). The interaction between frequency and epoch was significant (*F* (3, 225) = 10.827, *p* < 0.001, $\eta_{p}^{2}$ = 0.126). Moreover, a simple effect analysis showed that there was no significant difference in the RTs between high-frequency and low-frequency triplets at epoch one (*p* = 0.666). In epoch two, epoch three, and epoch four, the RTs of the random high-frequency triplets was significantly faster than that of the random low-frequency triplets (*p* < 0.001). The interactions between frequency and group (*p* = 0.299), as well as epoch and group (*p* = 0.934), were not significant. The interaction between frequency, epoch, and group was marginally significant (*F* (6, 225) = 1.872, *p* = 0.087, $\eta_{p}^{2}$ = 0.048). A simple effect analysis showed that there was no significant difference in the RTs between random high-frequency triplets and random low-frequency triplets (*p_s_* > 0.05) during epoch one of the three groups. During epoch two in the individual group and the cooperative group, there was also no significant difference in the RTs between the random high-frequency triplets and random low-frequency triplets (*p_s_* > 0.05). Nevertheless, during epoch two in the competitive group, the RT of the random high-frequency triplets was significantly faster than that of the random low-frequency triplets (*p_s_* < 0.05). Furthermore, during epoch three and epoch four in all three groups, the RT of the random high-frequency triplets was significantly faster than that of the random low-frequency triplets (*p_s_* < 0.05).

**Figure 6:**
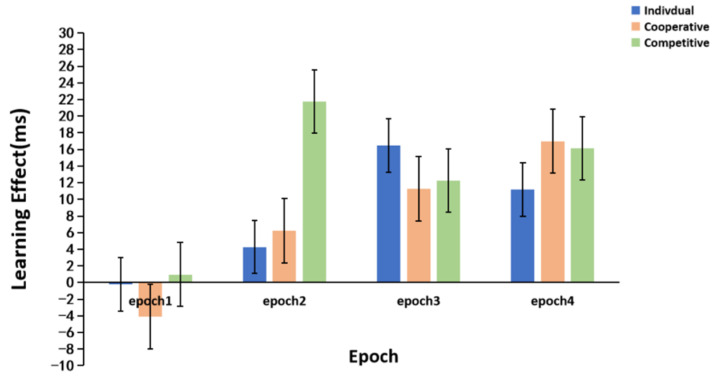
Statistical learning effects for the individual group, cooperative group, and competitive group. The error bars indicate the standard error.

The statistical learning effect was calculated as the median RT of the random low-frequency triplets, minus that of the random high-frequency triplets for each epoch, in each group, and the results are shown in Figure 6. The statistical learning effect for each group was analyzed by ANOVA. The results demonstrated that the main effect of epoch was significant (F (3, 225) = 10.827, *p* < 0.001, $\eta_{p}^{2}$ = 0.126). In addition, post-hoc comparisons indicated that the effect of epoch one was significantly less than that of epoch two, epoch three, and epoch four, but the differences between epoch two, epoch three, and epoch four were not significant. The main effect of the group was not significant (*F* (2, 75) = 1.228, *p* = 0.299, $\eta_{p}^{2}\;$= 0.032). Meanwhile, the interaction between epoch and group was marginally significant (*F* (6, 225) = 1.872, *p* = 0.087, $\eta_{p}^{2}$ = 0.048). A simple effect analysis showed that there was no statistical difference in the learning effect among the three groups at epoch one, epoch three, and epoch four (*p_s_* > 0.05). In addition, in epoch two the statistical learning effect of the competitive group was greater than those of the individual (*p* = 0.002) and cooperative (*p* =0.006) groups, but there was no significant difference between the individual group and the cooperative group (*p* = 0.710).

## 4. Discussion

Learning in cooperative and competitive situations has been revealed to provide motivation and to improve academic performance [8,12,13,54,55]. However, it remains unknown whether a similar effect exists regarding motor sequence learning. We wanted to demonstrate—and did demonstrate—that just one belief or thought about cooperation or competition is enough to affect general skill learning and statistical learning in motor sequence learning.

The results showed that the overall RTs of the three groups decreased with each successive epoch, indicating that general learning occurred [16,40]. The RT for the random high-frequency triplets was faster than that for the random low-frequency triplets, revealing that statistical learning occurred [17,18,36,37,48]. The task selection showed to be reasonable. For general learning, cooperative and competitive situations could, indeed, make the students learn faster. Furthermore, there was no significant difference in the learning time between the cooperative and competitive groups. However, there was no significant difference in the learning effect among the three groups. For statistical learning, both the cooperative and competitive groups responded faster than the individual group to both the random high-frequency and random low-frequency triplets. The situation setting of this study was shown to be effective for learning. The results—that the three groups ultimately showed no significant difference in learning effects in general learning and statistical learning, but a shorter learning time in cooperative and competitive situations—illustrated that these two methods are better than the individual situation. In other words, the situation of cooperation or competition enabled the students to acquire approximately the same learning effect in a shorter time period, compared with the individual situation.

To investigate the early stages of the learning process, an additional analysis was conducted on the changes from epoch one to epoch two. A decrease in the overall RT from epoch one to epoch two indicated that general learning had already occurred at an early stage. However, there was no significant difference in the effect of general learning among the three groups, suggesting that competition and cooperation have no influence on the general learning effect. In addition, the statistical learning of the three groups occurred very early, but the RT of the competitive group was faster, and the effect value was greater than those of the other groups. Thus, the competitive situation had a specific effect on learning, which was reflected in the acceleration of the statistical learning process, but no similar effect on general learning was observed. The advantage that appeared in epoch two of the competitive situation disappeared in the later stages, which is probably due to the task ceiling effect.

It should be pointed out that all participants were stressed by the experimental instructions to respond as quickly and accurately as possible, but the students in the cooperative or competitive situation did respond faster. It can be tentatively speculated that the setting situation can influence the learning process more effectively than simple experimental instructions. The setting of the situation is speculated to induce intrinsic drive more than the experimental instructions.

Practice has proven that both cooperative and competitive situations can stimulate and maintain the interest and motivation of students to learn, and can strengthen the learning effect [8,12,55]. Therefore, in order to improve the learning effect, the guidance of the instructor, as well as placing an emphasis on the setting of the learning situation, should be considered. There were two major limitations of this study: the difficulty of the task to be learned and the length of the learning process. These should be comprehensively taken into consideration when devising a learning situation. Situation setting as a teaching strategy is effectively supported by the results of our study, but it should be pointed out that the ecological validity of this study may require further improvement.

## 5. Conclusions

In summary, the current study showed, for the first time, that both cooperative and competitive situations have a positive effect on statistical learning. In this study, cooperative and competitive situations had a shortened learning time, compared to individual situations, but there was no significant difference in the learning effect among the three situations. Specifically, a competitive situation accelerated the statistical learning process but not the general learning process. The results suggest that cooperation and competition situations should be created in motor skill teaching to upgrade the quality of teaching. It is worth noting that the participants performed the experiment in pairs in the present study, but more participants per group could be considered in the future. Future research could also explore the impact of cooperative and competitive situations in real-world teaching practices. Furthermore, in future studies event-related potentials (ERPs) and functional MRI could be employed to explore the neural mechanisms of statistical learning enhancement.

## Data Availability

The original contributions presented in the study are included in the article, and further inquiries can be directed to the corresponding author.

## Supplementary Materials

The following supporting information can be downloaded at: https://www.mdpi.com/article/10.3390/brainsci12081059/s1, Figure S1: The key rules of ASRT task; Experimental task and situation instructions.
