# Supplementary material for: The Effects of Cooperative and Competitive Situations on Statistical Learning
Source: Brain Sci. 2022 Aug 10;12(8):1059. doi: 10.3390/brainsci12081059 (PMC9405654; doi:10.3390/brainsci12081059)
Supplement: Supplementary file 1 [file brainsci-12-01059-s001.zip › brainsci-1790859-supplementary.pdf]

Before the experiment began, the paired participants came into the lab at the same time, and each pair of participants was arranged in a dimly lit room. The two participants were separated by an opaque white partition and were required to be quiet during the experiment. Each participant sat comfortably about 60 cm in front of a computer screen.

Instruction:

1、Experimental task instruction:

Welcome to our experiment!

First, a "+" fixation appears in the center of the computer screen to remind you to start the experiment. Next, four squares are presented, and you should respond as quickly and accurately as possible to the keys corresponding to the red squares.

Please sit down and put your hands on the keyboard. Place your left index finger on the "X" key and your left middle finger on the "Z" key. Place your right index finger on the "N" key and your right middle finger on the "M" key. You need to respond with the corresponding keys. The ASRT task has a total of 20 blocks, so rest for at least 20 seconds before starting the next block. The whole process takes about 20 minutes.

When you're ready, press the SPACEBAR to start the formal experiment.

The key rules are shown in the figure:

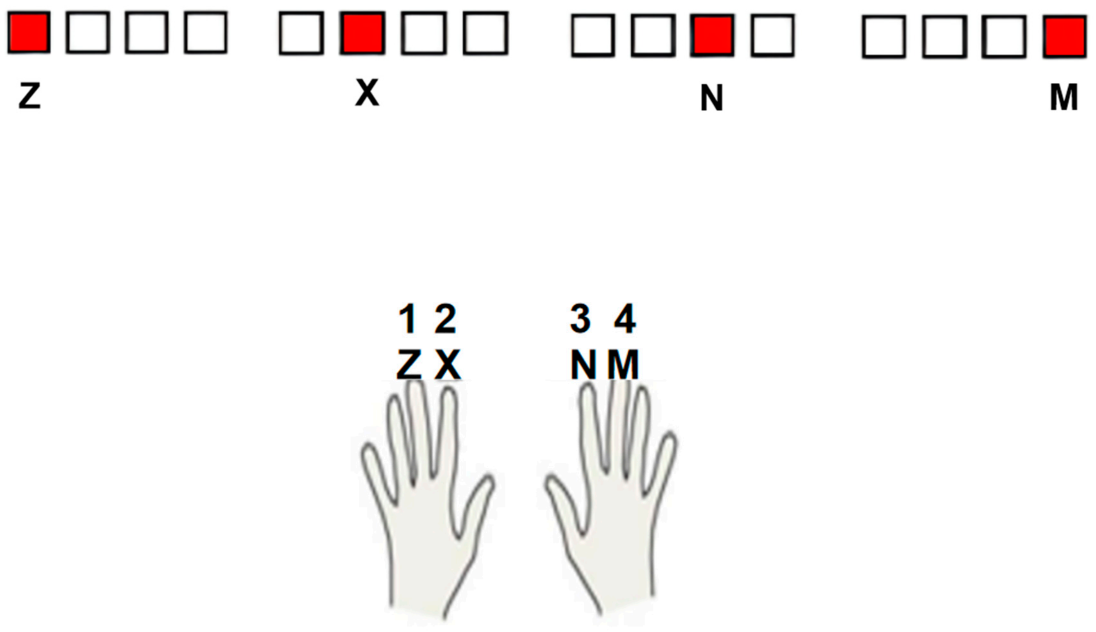

Figure S1. The key rules of ASRT task.

2、Experimental situation instruction:

Participants were told about the situation they were in.

The instructions for the individual situation group:

You need to complete the task on your own, and that you have no role relationship with your paired participant. You need to focus on improving your score, and your score will display on the screen after each Block is completed, without being seen by your paired participant.

The instructions for the cooperative situation group:

You and your paired participant are cooperative relationships, and you need to work with your partner to improve the total score both of you. This score is the total score you and your partner performed, and will present on your and your partner's computer screen at the end of each block.

The instructions for the cooperative situation group:

You and your paired participant are opponent relationships, and you need to try to exceed your opponent's score. This score is determined based on your performance, and at the end of each block, your and your opponent's scores will present on your and your opponent's computer screens.

At the end of each block you need to take a break of about 20 seconds to avoid fatigue during the experiment.
